# Retraction: Analysis the patients’ careflows using process mining

**DOI:** 10.1371/journal.pone.0357078

**Published:** 2026-08-27

**Authors:** The PLOS One Editors

After this article [1,2] was published, the following concerns were noted:

- The article reports the use of clinical data, but provides no information regarding ethics approval or consent for the use of this data,
- The article does not provide sufficient information about the source of the clinical data or the number of individuals whose data was used for this study.

The third author stated that they were unable to provide documentation to confirm that ethics approval was obtained for the use of this data.

In addition, the Editors have concerns about the integrity and/or reliability of the peer review process. The Editors do not have concerns regarding the authors’ conduct during the peer review process.

The *PLOS One* Editors retract this article [1,2] due to concerns about compliance with PLOS policy on Human Subjects Research.

In light of the matters noted above, PLOS will not provide an update of the Data Availability statement.

AHMR, DSA, and MA did not agree with the retraction. NEEA either did not respond directly or could not be reached.
